# Arch Coombs Hart, Ph.B., D.D.S., M.D.

**Published:** 1901-10

**Authors:** 


### Obituary.

### ARCH COOMBS HART, Ph.B., D.D.S., M.D.

Dr. Hart died at his residence in San Francisco, Cal., May 28, 1901, of Addison's disease and acute cerebral hyperæmia.

He was born in China, Me., January 14, 1869; graduated from the University of the Pacific in 1889, from the Department of Dentistry, University of Pennsylvania, in 1892, and from the Medical Department of the College of Physicians and Surgeons, San Francisco, in 1897.

He commenced practice at Pacific Grove in 1892 and moved to San Francisco in November of that year, where he remained until his death. He was lecturer on stomatology in the Hahnemann Hospital College at the time of his death. He also held the office of corresponding secretary in the California State Dental Association.

The *Pacific Dental Gazette* says of him: "Dr. Hart was a man of untiring energy, enthusiastic in his profession, and endowed with a mind which would not permit him to follow beaten paths, but led him to study along lines of original research in the various departments of dental science."

It seems peculiarly fitting that after well-spent lives the aged should pass on to other conditions, but it is impossible to feel reconciled to the loss of the young. Dentistry has added, in the past few years, many young men and women of thorough education to its ranks, with the result that its literature has broadened and the scientific value of its work is steadily on the increase. When, therefore, death invades the ranks of the younger generation, as has been the case in several instances in recent years, the loss is deeply felt. Dr. Hart had the enthusiasm of youth. He was an untiring worker, and doubtless exhausted his vitality by his constant efforts.

While his deductions from his investigations were not always in accordance with accepted opinions, and perhaps not always sufficiently proved to be acceptable, yet the energy displayed in continuing these investigations gave promise of rich fruitage in the future.

His independence of character was manifested in working his way through his dental studies without material aid from any one. To the writer he frequently expressed his satisfaction with this experience, and doubtless its necessary privations helped to strengthen his character for the serious work to which he had devoted his life.

While many were unable to understand Dr. Hart, and in many instances failed to appreciate his worth, there were more who knew his underlying strength of character, and anticipated that in the development of years much would be accomplished.

His death should carry with it a warning. The effort to accomplish in a few years that which should cover a lifetime has been the rock upon which many a life barque has been sacrificed.

Dr. Hart was married to Nella R. Lawrence in San Francisco, September 5, 1894. Two children, a son and a daughter, survive him.

Resolutions on the death of Dr. A. C. Hart, adopted by the Oakland Dental Club, June 5, 1901:

The Oakland Dental Club desire to express their great sorrow and deep regret at the untimely death of Dr. A. C. Hart, of San Francisco. The sudden ending of this life, freighted with so much promise, comes as a calamity to the dental profession at large, and fills every member of the Club with a profound sense of personal loss. The ending of a life which has run the due course of time attached to man in this world, and which has been filled with the largest measure of usefulness to humanity, may be looked upon as a benediction, but our friend who has been so suddenly removed from earthly environment was but upon the threshold of a career which has not only already enriched science, but gave vigorous promise of much larger contribution from his continued research, together with the added power of a maturer mind, and we cannot therefore but grieve at the abrupt termination of a life so auspiciously begun. Be it therefore

*Resolved*, That in the death of Dr. Hart the dental profession has lost one of its ablest, keenest, and most energetic investigators.

